# Impact of telelactation services on breastfeeding outcomes among Black and Latinx parents: protocol for the Tele-MILC randomized controlled trial

**DOI:** 10.1186/s13063-021-05846-w

**Published:** 2022-01-03

**Authors:** Lori Uscher-Pines, Jill Demirci, Molly Waymouth, Rebecca Lawrence, Amanda Parks, Ateev Mehrotra, Kristin Ray, Maria DeYoreo, Kandice Kapinos

**Affiliations:** 1 RAND Corporation, 1200 S Hayes St, Arlington, VA 22202 USA; 2 University of Pittsburgh School of Nursing, 3500 Victoria Street, Pittsburgh, PA 15261 USA; 3 RAND Corporation, 4570 Fifth Avenue, Pittsburgh, PA 15213 USA; 4 Virginia Commonwealth University, 806 W. Franklin St., Richmond, VA 23284-2018 USA; 5 Harvard Medical School, 180 Longwood Avenue, Boston, MA 02115 USA; 6 University of Pittsburgh, 3414 Fifth Avenue, Pittsburgh, PA 15213 USA; 7 RAND Corporation, 1776 Main Street, Santa Monica, CA 90401-3208 USA; 8 RAND Corporation and University of Texas Southwestern Medical School, 1200 S Hayes St, Arlington, VA 22202 USA

**Keywords:** Randomized controlled trial, Breastfeeding, Telehealth, Telelactation, Health equity, Digital trial

## Abstract

**Background:**

Breastfeeding offers many medical and neurodevelopmental advantages for birthing parents and infants; however, the majority of parents stop breastfeeding before it is recommended. Professional lactation support by the International Board Certified Lactation Consultants (IBCLCs) increases breastfeeding rates; however, many communities lack access to IBCLCs. Black and Latinx parents have lower breastfeeding rates, and limited access to professional lactation support may contribute to this disparity. Virtual “telelactation” consults that use two-way video have the potential to increase access to IBCLCs among disadvantaged populations. We present a protocol for the digital Tele-MILC trial, which uses mixed methods to evaluate the impact of telelactation services on breastfeeding outcomes. The objective of this pragmatic, parallel design randomized controlled trial is to assess the impact of telelactation on breastfeeding duration and exclusivity and explore how acceptability of and experiences with telelactation vary across Latinx, Black, and non-Black and non-Latinx parents to guide future improvement of these services.

**Methods:**

2400 primiparous, pregnant individuals age > 18 who intend to breastfeed and live in the USA underserved by IBCLCs will be recruited. Recruitment will occur via Ovia, a pregnancy tracker mobile phone application (app) used by over one million pregnant individuals in the USA annually. Participants will be randomized to (1) on-demand telelactation video calls on personal devices or (2) ebook on infant care/usual care. Breastfeeding outcomes will be captured via surveys and interviews and compared across racial and ethnic groups. This study will track participants for 8 months (including 6 months postpartum). Primary outcomes include breastfeeding duration and breastfeeding exclusivity. We will quantify differences in these outcomes across racial and ethnic groups. Both intention-to-treat and as-treated (using instrumental variable methods) analyses will be performed. This study will also generate qualitative data on the experiences of different subgroups of parents with the telelactation intervention, including barriers to use, satisfaction, and strengths and limitations of this delivery model.

**Discussion:**

This is the first randomized study evaluating the impact of telelactation on breastfeeding outcomes. It will inform the design and implementation of future digital trials among pregnant and postpartum people, including Black and Latinx populations which are historically underrepresented in clinical trials.

**Trial registration:**

ClinicalTrials.gov NCT04856163. Registered on April 23, 2021

## Administrative information

Note: the numbers in curly brackets in this protocol refer to SPIRIT checklist item numbers. The order of the items has been modified to group similar items (see http://www.equator-network.org/reporting-guidelines/spirit-2727-statement-defining-standard-protocol-items-for-clinical-trials/).

**Table:** 

Title {1}	Impact of telelactation services on breastfeeding outcomes among Black and Latinx parents: protocol for the Tele-MILC randomized controlled trial
Trial registration {2a and 2b}.	ClinicalTrials.gov NCT04856163
Protocol version {3}	Version 1, 09.12.2021
Funding {4}	This trial was funded by a grant from the National Institutes of Health (R01NR018837)
Author details {5a}	Lori Uscher-Pines, PhD, MSC^1^; Jill Demirci, PhD, RN, IBCLC^2^; Molly Waymouth^1^; Rebecca Lawrence^3^; Amanda Parks^4^; Ateev Mehrotra, MD, MPH^5^; Kristin Ray, MD, MS^6^; Maria DeYoreo, PhD^7^; Kandice Kapinos, PhD^1^ 1 RAND Corporation, Arlington VA 2 University of Pittsburgh School of Nursing, Pittsburgh PA 3 RAND Corporation, Pittsburgh PA 4 Virginia Commonwealth University, Richmond VA 5 Harvard Medical School, Boston MA 6 University of Pittsburgh, Pittsburgh PA 7 RAND Corporation, Santa Monica CA
Name and contact information for the trial sponsor {5b}	National Institute of Nursing Research (NINR) within the National Institutes of Health (NIH). Contact sungsug.yoon@nih.gov]
Role of sponsor {5c}	The funder had no role in the study design, data collection, data analysis, data interpretation, or writing.

## Introduction

### Background and rationale {6a}

Breastfeeding offers numerous medical and neurodevelopmental advantages for birthing parents and infants, with increased benefits with longer breastfeeding duration [1–4]. Breastfeeding includes feeding directly at the breast/chest as well as the provision of human milk (e.g., through pumping or expressing milk). Unfortunately, the majority of parents in the USA stop breastfeeding before it is recommended, with fewer than half of infants receiving any breastmilk at 6 months of age [5]. Breastfeeding rates are even lower among minoritized communities; for example, only 30% of Black infants receive breastmilk at 6 months [6]. Professional lactation support by the International Board Certified Lactation Consultants (IBCLCs) has been shown to improve breastfeeding duration and exclusivity (i.e., proportion of breastmilk in an infant’s diet) [7–10]. As such, the *Surgeon General’s Call to Action to Support Breastfeeding* identifies increasing access to IBCLCs as a policy priority [11]. IBCLCs provide dedicated management of lactation problems that other providers (e.g., pediatricians) may not address because of limited training and competing demands [12, 13]. Poor access to IBCLCS among minoritized and rural parents may contribute to breastfeeding disparities [14].

Telelactation video visits may increase access to IBCLC services. IBCLC services are traditionally delivered in-person; however, telelactation, which allows remote IBCLCs to connect with breastfeeding parents via two-way video, is a potential alternative. Telelactation is less costly and more convenient than in-person office visits and allows new parents to avoid traveling with their infants. It can also address healthcare professional shortages, bringing IBCLCs into communities that lack them. In prior observational studies and case reports, telelactation was offered to augment in-person lactation support within the medical home or at IBCLC offices. Although informative and demonstrating promise, prior studies were small, used outdated technology, and assessed indicators of satisfaction and convenience rather than impacts on breastfeeding rates [15–19]. Multiple companies [20] now offer a model of telelactation where patients can initiate video calls with lactation consultants using their personal devices (e.g., mobile phones) and access support on-demand at any time of day. The COVID-19 pandemic further accelerated the use of telelactation, with providers increasingly turning to virtual visits to maintain access to professional lactation support and avoid exposure to COVID-19. Despite the proliferation of these services, very little is known about the effect of telelactation. To date, no definitive evidence exists on whether these services improve breastfeeding rates and whether they are feasible and acceptable to different populations of parents.

In a pilot randomized trial of telelactation [21], our study team demonstrated the feasibility of on-demand telelactation and showed it was highly valued by new parents [22–26]. Increased breastfeeding duration was observed among participants receiving telelactation, but the sample size was small and differences were not statistically significant. Also, the pilot occurred in a single, predominantly White community in rural Pennsylvania. A larger, more diverse study population is needed to quantify the impact of telelactation on breastfeeding outcomes, including the effects on subpopulations with historically low breastfeeding rates. The Tele-MILC (Telehealth for Mothers to Increase Lactation Confidence) trial described in this study protocol is designed to address this gap.

### Objectives {7}

The trial has two aims and several sub-aims. The first aim is to assess the impact of telelactation on breastfeeding duration and exclusivity. Sub-aim 1A is to assess the effect of telelactation on breastfeeding outcomes across Latinx, Black, and non-Black and non-Latinx (predominantly White) parents and across rural and urban parents. Sub-aim 1B is to explore whether breastfeeding self-efficacy is a mediator for any observed improvements in breastfeeding outcomes. We hypothesize that a higher proportion of individuals with access to telelactation will be (1) breastfeeding at all and [2] breastfeeding with less formula use at 24 weeks postpartum and that these higher breastfeeding rates will be mediated by increases in breastfeeding self-efficacy. The second aim is to explore how acceptability of and experiences with telelactation vary across Latinx, Black, and non-Black and non-Latinx parents to guide improvements in these services.

### Trial design {8}

The trial is a pragmatic, parallel-design randomized controlled trial. It is a digital trial, with all activities from recruitment to intervention delivery and outcome assessment conducted online. The overall study design is mixed methods and uses a sequential explanatory design in which qualitative interviews are used to explain and contextualize findings from the quantitative outcomes analysis.

## Methods: participants, interventions, and outcomes

### Study setting {9}

In this fully digital trial, there are no physical study sites; however, study team members are located at RAND, University of Pittsburgh, and Harvard University. Recruitment occurs via Ovia’s pregnancy tracker mobile phone app. The Ovia app is one of the most popular pregnancy apps available for free download [27]. Used by over 2 million people in the USA each month, it provides educational content, conducts health assessments, and uses proprietary algorithms and machine learning to provide user-specific support, advice, and resources [27]. Study participants are drawn from a large community of active Ovia app users across the USA. Our focus will be on the subset of Ovia users residing in 39 US states and territories with fewer than 5 IBCLCs per 1000 births [28]. However, if recruitment occurs more slowly than planned, we will expand recruitment to Ovia users in all US states, and we will add Facebook as a recruitment site. With Facebook, we will contact the administrators of pregnancy and parenting groups and ask them to advertise the study to group members on our behalf.

### Eligibility criteria {10}

Eligible parents include those who are (1) ≥ 18 years of age, (2) pregnant with their first child, (3) intending to attempt breastfeeding, and (4) residing in a state underserved by IBCLCs. Exclusion criteria include (1) non-singleton pregnancy, (2) advised by a healthcare provider not to breastfeed for a medical reason (e.g., HIV+ status, chemotherapy planned), (3) police custody or incarceration, and (4) infant to be separated from gestational parent (e.g., given up for adoption, surrogate, protective custody).

### Who will take informed consent? {26a}

All potential trial participants provide informed consent in an eConsent process. The eConsent process features a short slide show which summarizes key points, a long form (detailed) consent statement, and an interactive quiz to probe understanding. The quiz is included to reinforce key concepts. Participants who enter incorrect answers are shown the correct answer with an explanation. Because incorrect answers receive this correction and reinforcement, incorrect answers do not prevent participants from continuing with consent and participating in the study. Participants sign the long form consent statement once they have reviewed all materials to their satisfaction and agree to participate.

### Additional consent provisions for collection and use of participant data and biological specimens {26b}

There are no additional consent provisions because this is not applicable.

### Interventions

#### Explanation for the choice of comparators {6b}

Eligible participants will be randomized into one of two study arms: (1) telelactation services via on-demand video visits on personal devices and [2] usual care (i.e., without telelactation services). Participants in the intervention arm receive access to a telelactation mobile phone application, and those in the usual care (control) arm receive an ebook on newborn care published by the American Academy of Pediatrics. The ebook is provided for equipoise so that participants in both study arms receive a parenting resource of value through the study. Because the ebook has limited breastfeeding content, it should not significantly influence breastfeeding behaviors or rates.

#### Intervention description {11a}

##### Intervention arm

Participants randomized to the intervention arm receive unlimited, on-demand telelactation visits through Pacify’s mobile phone app, which is available for download on both Apple and Android devices. After consent, participants in the intervention arm receive an email that provides a complete orientation to the Pacify app (e.g., technical requirements, how to download the app), and they receive a unique coupon code that they can enter to unlock unlimited free visits starting at the time of consent and extending for 6 months past their due date.

Pacify IBCLCs are available 24 h a day and typically answer calls within seconds of a visit request. The app can support visits in English or Spanish. To use the Pacify app, participants need a smartphone or tablet. Participants also need access to Wi-Fi or a cellular network to initiate a visit.

##### Usual care (control) arm

Participants in the control arm receive care as usual. Although they are not exposed to IBCLCs within the context of the study, they may receive IBCLC or other lactation support from other sources. They also have access to the limited breastfeeding content within the Ovia pregnancy app, including peer support though community boards. In addition, participants in this arm receive an ebook on infant care published by the American Academy of Pediatrics. After consent, participants in this arm receive an email explaining how to access their ebook.

#### Criteria for discontinuing or modifying allocated interventions {11b}

Informed consent procedures clarify that participants can withdraw from the study at any time for any reason. Also, participants can decide whether when and how to use the free parenting resources (telelactation app or ebook) provided by the study.

#### Strategies to improve adherence to interventions {11c}

Participants in the telelactation arm are incentivized to download the app, set up a Pacify account, and initiate a test call within 2 weeks of enrollment, and participants in the care as usual arm are incentivized to access their ebook. Participants who set up Pacify account (telelactation arm) or redeem their ebook (usual care) receive a $20 gift card.

With the support of Pacify, we track app downloads and account creation, with the goal of having 75% of telelactation arm participants with a working Pacify account by their due date. Those participants who do not download the app on their own within 2 weeks are sent reminders. Further, throughout the study period, telelactation arm participants receive text messages and push notifications from Pacify reminding them that the app is available for use. While use of the app is encouraged, the choice to use telelactation is the participant’s. Participants also exercise full discretion on the frequency and timing of visits.

#### Relevant concomitant care permitted or prohibited during the trial {11d}

This is a pragmatic trial. There are no limitations on care, and no care is prohibited.

#### Provisions for post-trial care {30}

Telelactation arm participants can continue to use the telelactation app after the trial has ended. Access to Pacify remains free for up to 2 years.

#### Outcomes {12}

The primary outcomes focus on breastfeeding duration and exclusivity at 6 months postpartum. Breastfeeding duration will be assessed in two ways: (1) any (self-reported) breastfeeding at 6 months (yes/no) and (2) time (in months) to cessation of breastfeeding as measured by reported age of infant when they completely stopped receiving the participant’s own milk. Breastfeeding exclusivity is defined as no formula use in the prior 24 h (yes/no) as reported at 6 months postpartum. Secondary outcomes include breastfeeding satisfaction (among parents who continue to breastfeed) as self-reported at 6 months postpartum. To assess breastfeeding satisfaction, we will use the five-item maternal/infant breastfeeding satisfaction scale, which is a subscale of the H&H lactation scale [29]. An additional secondary outcome is experience with telelactation. This will be assessed using qualitative data from semi-structure interviews with telelactation arm participants.

#### Participant timeline {13}

**Fig. 1:**
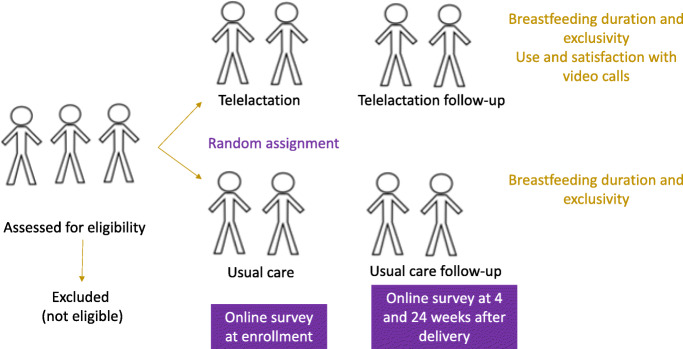
Trial process

To recruit participants from the population of Ovia app users, we designed a set of advertisements directing potential participants to a secure study website maintained by Datstat [30], a widely used platform that facilitates secure data collection. Potential participants who visit the study website after clicking on a study ad are directed to complete an online eligibility screening survey (Fig. 1). Those who are eligible based on their responses are directed to an eConsent process where they are asked to indicate whether they agree to participate in the study and to provide contact information. Those who consent are randomized to receive telelactation services or usual care. At the time of enrollment, all participants complete an online baseline survey. At enrollment, they indicate their due date and then receive text messages and emails to complete additional online surveys 4 weeks after their due date and 24 weeks after their delivery date (as self-reported in the 4-week survey). Further, we estimate 50 telelactation arm participants will be invited to participate in semi-structured interviews with the study team to explore experiences with telelactation. Interviews will be conducted at 8–12 weeks postpartum.

### Sample size {14}

We plan to recruit 2400 participants over 27 months. To meet this target sample size, we first estimated the number of study participants needed to achieve 80% power in the primary, intent-to-treat (ITT) analysis, assuming a type 1 error rate of 5%. We applied national estimates of breastfeeding rates among diverse groups who had initiated breastfeeding and estimated that approximately 50–60% of control group participants would be breastfeeding at 24 weeks [31]. To detect a clinically significant difference of 10-percentage points in any breastfeeding at 24 weeks postpartum across the study arms (telelactation vs. usual care), we would need 355–390 participants in each study arm (710–780 participants total) if using either a test of equal proportions or testing significance of the “treatment” variable in a logistic regression model. This effect size corresponds to a clinically significant effect size within the range of published effect sizes for IBCLC interventions [10]. It is also within the range we expect to see given certain planned changes to the study protocol relative to the pilot study, which had between–group differences in rates of breastfeeding at 3 months post-partum of 3–11%.

Next, because we also plan to examine whether intervention effects vary across specific racial and ethnic groups (Black, Latinx, and non-Black and non-Latinx) and for rural vs. urban participants, we estimated the number of participants needed for this aim. For this analysis, we will fit a logistic regression model that includes the treatment dummy and a vector of race dummy variables, as well as their interactions. We assumed that breastfeeding rates at 24 weeks among control group participants are consistent with current CDC estimates (62% for non-Latinx and non-Black, 59% for Latinx, and 53% for Black participants [31]) and that we will secure equal numbers of participants in each combination of race/ethnicity and treatment. To achieve statistical power of 80% to detect heterogeneous treatment effects by race/ethnicity (e.g., effect size of 15% for Black, 15% for Latinx, and 0% for non-Black and non-Latinx participants; or effect size of 0% for Black, 0% for Latinx, and 15% for non-Black and non-Latinx participants), we would require 1800 participants. After inflating the sample size to account for 25% dropout, which is common for digital trials [32, 33], we would need to enroll approximately 2400 participants (800 in each subgroup). This sample size provides 80% power to detect an overall effect size as small as 6.5% across arms. If our final sample is representative of Ovia users in terms of rural vs. urban distribution, then 2400 participants will also provide sufficient power for testing whether treatment effects differ by rural vs. urban location. These sample sizes provide sufficient statistical power (>80%) for our additional planned analyses, including detecting the treatment effect in the instrumental variable analysis (which has similar sample size requirements) [34] and an additional survival analysis within Aim 1. Further, because the inclusion of covariates generally increases power, these estimates are conservative given the analysis plan.

### Recruitment {15}

**Fig. 2:**
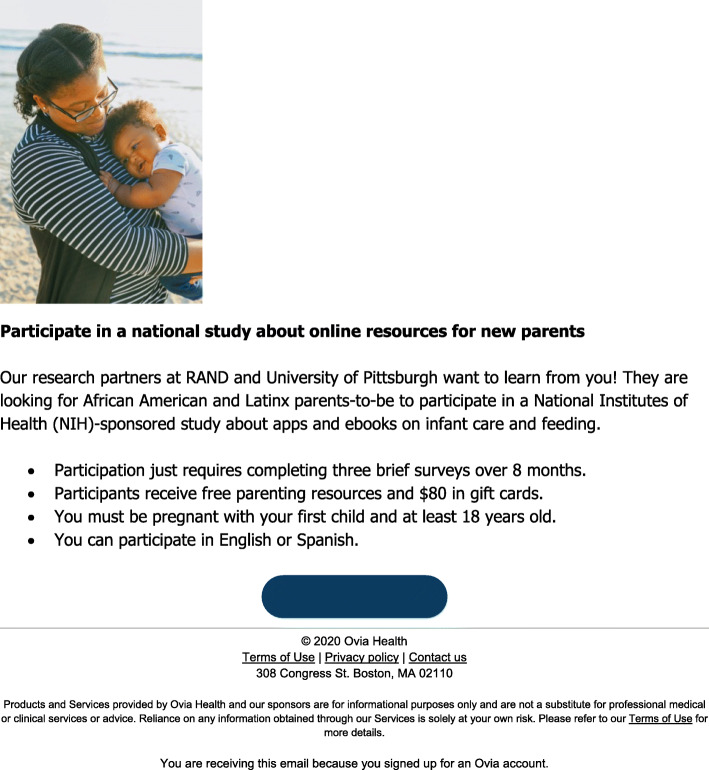
Sample study ad

Ovia sends emails featuring recruitment ads to subsets of the eligible population up to 3 times per month, and ads also appear in the within-app timeline of potentially eligible users. Ads we developed (Fig. 2), as well as all other study materials including draft surveys, were reviewed by a diverse advisory group of 18 parents who were recruited for this advisory group via Ovia. Materials were modified based on advisory group feedback prior to fielding.

Ads will run over 27 months and will be periodically suspended to pace recruitment (e.g., allow for participant verification). Throughout the recruitment period, we conduct ongoing monitoring for cases of fraudulent enrollment (e.g., bots rather than humans completing surveys, ineligible participants misrepresenting themselves, eligible participants attempting to enroll more than once) and investigate suspicious cases.

### Assignment of interventions: allocation

#### Sequence generation {16a}

Participants are randomized by the Datstat system in a 1:1 ratio using block randomization stratified by race (White, Black, Latinx, Other). The eligibility screening survey includes two questions on race and ethnicity that are used for randomization. If a participant indicates that they identify as Latinx, they are automatically categorized as Latinx regardless of how they respond to the question on race. Further, if a participant indicates that they do not identify as Latinx for their ethnicity and they identify as Black race, they are categorized as Black even if they select other races in addition to Black. Participants who indicate that they do not identify as Latinx and choose White race (only) are categorized as White. Participants who indicate that they do not identify as Latinx and choose one or more races other than White (e.g., Asian) or White and some other race(s) are categorized as Other.

We prepared a randomization tool which uses randomly drawn numbers to assign participants to a study arm following consent. Following randomization and group allocation, participants are directed to the baseline survey and provided with instructions relevant to their study arm.

#### Concealment mechanism {16b}

Because randomization occurs using an online tool (i.e., Datstat has programmed its system to pull from spreadsheets of random numbers to determine assignment), there is no human involvement, and the process is fully concealed from both study investigators and prospective participants until the study arm is assigned.

#### Implementation {16c}

This is not applicable because this is fully digital trial.

### Assignment of interventions: blinding

#### Who will be blinded {17a}

While it is not feasible to blind participants to assignment post-enrollment, IBCLCs delivering telelactation are not aware of who is a study participant vs. a member of the general population of Pacify users from across the USA.

#### Procedure for unblinding if needed {17b}

This is not applicable because participants will not be blinded.

### Data collection and management

#### Plans for assessment and collection of outcomes {18a}

Study data with the exception of semi-structured interviews are collected through online surveys and rely heavily on previously used and well-validated instruments that take an agnostic approach with respect to infant feeding. All enrollment materials and surveys are available in English and Spanish.

At enrollment, all participants complete a baseline survey. The baseline survey encompasses an assessment of demographics as well as participants’ baseline breastfeeding intentions and attitudes. Further, it addresses digital and health literacy, trust in technology, and employment plans. Later assessments (4 weeks and 24 weeks post-partum) capture study outcomes (e.g., duration of breastfeeding, amount of formula) and uptake of the intervention (e.g., use of telelactation). The 4-week survey covers the following additional topics: birth experiences, lactation support received, breastfeeding self-efficacy, breastfeeding challenges, mental health, and COVID-19 vaccination. The 24-week survey covers the following additional topics: breastfeeding challenges, breastfeeding satisfaction, use of donor breast milk, breastfeeding support received, intention to breastfeed additional children, trust in healthcare providers, experiences with discrimination in healthcare, work status, and social support.

Surveys take 15–20 min to complete depending on study arm and breastfeeding status. Participants receive $20 gift cards to either Amazon or Walmart for each assessment (baseline, 4 weeks, and 24 weeks) and $20 for completing the step required for their study arm (download of Pacify app or ebook), for a total incentives value of $80.

Pacify also supplies data to the study team on app utilization (e.g., app downloads, number of telelactation visits, timing of visits, issues discussed). These data will be used in combination with survey data to assess use (including dose) of the intervention.

For aim 2, we will interview approximately 50 participants randomized to receive telelactation. Participants will be purposively sampled to explore how different populations (Black, Latinx, non-Black, and non-Latinx, rural, urban) experience telelactation. Within each subgroup, we will seek maximum diversity with respect to telelactation use (e.g., no use, frequent use). Participants will be invited to participate between 8 and 12 weeks postpartum and will receive a $25 gift card as an incentive to participate. We will use a semi-structured interview protocol to ensure key questions are addressed and to permit comparisons across groups. Participants will be asked to describe their experience and satisfaction with telelactation, reasons for and barriers to use, cultural appropriateness, and the strengths and limitations of these services. We will also ask about their general experiences with breastfeeding and other forms of support received. Survey instruments and interview guides will be included in appendices in peer-reviewed publications if permitted and made available upon request.

#### Plans to promote participant retention and complete follow-up {18b}

The trial incorporates multiple strategies to promote retention. First, participants receive an incentive for each survey that they complete. Second, after an initial email inviting participants to complete each survey, we send additional text and phone call reminders to those who do not complete a scheduled assessment within 3 days. Non-responders receive up to three reminder emails, two text messages, and one phone call per survey. For those participants who do not complete the final (24-week survey), we also contact a family member (contact information provided during the consent process along with permission to contact) and ask them to remind the participant to complete the survey. Third, we have incorporated a variety of strategies to promote ongoing interest and engagement in the study. We send newsletters to active participants and run a variety of contents and games (e.g., photo contest, trivia contest) in which one winner is selected and receives a small incentive. We also created a short video featuring members of the study team to thank participants for their participation. This video is designed to tap into participants’ altruism and the role they are playing in contributing to science and to personalize the study, demonstrating that there are real people behind it.

#### Data management {19}

Study data is collected and managed through Datstat, a secure, encrypted data management system [30]. Participants complete all assessments online and send questionnaire data securely through Datstat. Datstat’s platform operates on a protected server. Data stored on Datstat’s platform is accessed by select members of the study team only, through a secure, password protected portal.

#### Confidentiality {27}

Personal information is collected during the consent process (e.g., name, email, phone number) and in surveys. The survey data files only have unique study IDs and do not contain contact or identifying information. A single password-protected, user-restricted file links participants’ study IDs to their names and contact information. This participant identification crosswalk is stored on Datstat’s encrypted server.

#### Plans for collection, laboratory evaluation and storage of biological specimens for genetic or molecular analysis in this trial/future use {33}

This is not applicable because the study does not collect biological specimens.

### Statistical methods

#### Statistical methods for primary and secondary outcomes {20a}

To assess the impact of telelactation, we will estimate the intervention effect sizes for primary binary outcomes including (1) any breastfeeding at 24 weeks and (2) exclusive breastfeeding at 24 weeks. Our key independent variable will be an indicator variable equal to one for participants randomized to receive telelactation and zero otherwise, but we will also use a count measure of the number of telelactation visits to test whether there is a dose-response effect. We will use an intention-to treat (ITT) approach as the primary approach to estimate the effect of the treatment [35]. We will calculate and compare unadjusted means and associated confidence intervals of our outcome variables testing for statistical differences across the intervention and control group. Next, we will fit logistic regression models for each binary outcome to account for covariates that may be predictive of the outcomes or potential confounders in the regression.

We will also look at breastfeeding duration as a time-to-event variable with the event being defined as cessation of all breastfeeding. Time to cessation is measured in weeks as of the 24-week survey and can be modeled using survival analytic (time to event) methods. In the Cox proportional hazards (CPH) model [36], the measure of effect is the hazard rate, which here is the probability of cessation up until the point of measurement (e.g., 4 weeks, 24 weeks). The hazard ratio can be used to compare the study arms, giving the ratio of the total number of observed to expected cessations in the telelactation group relative to the usual care group.

We will explore the role of self-efficacy as a possible mediator for intervention and dose-response effects. Specifically, we will first establish whether treatment via telelactation is a significant predictor of breastfeeding self-efficacy (as measured among breastfeeding participants at 4 weeks), and separately whether self-efficacy is a significant predictor of the probability of breastfeeding. We will then estimate the simultaneous effects of treatment and self-efficacy on the probability of breastfeeding by including self-efficacy as an additional covariate in the logistic regression model described above and will assess the significance of treatment status and self-efficacy in predicting breastfeeding probability. If self-efficacy remains statistically significant after controlling for treatment, this suggests that some form of mediation is supported.

#### Interim analyses {21b}

The study team’s statistician will conduct an interim analysis 12 months into data collection. The purpose of this analysis is to (1) inform any design changes that may be needed (e.g., new strategies to limit attrition) and (2) inform the conduct of the qualitative interviews including the sampling strategy and interview protocol. These analyses will not be used to inform termination decisions because the interim analysis will not be powered to detect heterogeneity of treatment effects.

#### Methods for additional analyses (e.g., subgroup analyses) {20b}

We will determine whether the intervention and dose-response effects differ by race and ethnicity. We will use similar analytic approaches as described above controlling for key baseline covariates and main effects for treatment status, but also will include interaction terms between treatment status and race/ethnicity to determine the effects of the intervention for Black (projected to be 33% of final sample), Latinx (projected to be 33% of the final sample), and non-Black and non-Latinx (predominantly White) participants. We will also test for treatment effects by rural vs. urban location, using similar methods.

#### Methods in analysis to handle protocol non-adherence and any statistical methods to handle missing data {20c}

While traditional guidelines for randomized controlled trials (RCT) recommend the primary approach be ITT [37, 38], ITT ignores contamination, measuring the effect of assignment to treatment, rather than the effect of receiving a treatment. To maximize power and estimate the effect of receiving treatment, we will implement an approach specifically designed to address the issue of noncompliance to treatment in an RCT, called a contamination adjusted intent-to-treat (CA ITT) analysis [39]. We will use an instrumental variable model (IV), with treatment assignment as the “instrument.”

It is likely that some data needed for our analyses will be missing (e.g., incomplete covariates and some attrition). To avoid biasing the results or excluding data, we will impute missing values prior to analysis using multiple imputation methods. We will create multiple imputed complete datasets to account for the uncertainty in the missing values, and combine estimates from completed datasets using standard multiple imputation combining rules [40].

#### Plans to give access to the full protocol, participant level data, and statistical code {31c}

Data resulting from the proposed research will be shared with external researchers who request access beginning 1 year after the project ends. Results will be available to other researchers and practitioners following a brief application process that explains how the data will be used and protected. Constraints imposed by protection regulations for human research subjects (e.g., HIPAA Protected Health Information) and RAND’s Institutional Review Board (IRB) will be recognized as allowed by the NIH. External researchers interested in investigator data and other research methodology and procedures will obtain this information through collaborative agreements (e.g., data use agreements) with the principal investigator and co-investigators, as required by the NIH’s data sharing policy.

### Oversight and monitoring

#### Composition of the coordinating center and trial steering committee {5d}

The NIH and RAND’s Institutional Review Board determined that this trial does not meet criteria for the establishment of a data and safety monitoring board because it is a single (digital) site trial of a low-risk intervention. However, a subset of the study team meets weekly throughout the recruitment period to manage the trial. Data and safety monitoring is a component of regularly scheduled weekly meetings with the study team. Meetings involve a review of collected data (including adverse events, unanticipated problems, and subject withdrawals) to determine whether there is any change to the anticipated benefit-to-risk assessment of study participation and whether the study should continue as originally designed, should be changed, or should be terminated. At these meetings, the study team also discusses advances in research concerning breastfeeding support and telehealth. The principal investigator is ultimately responsible for participant safety and ongoing evaluation of the study’s progress.

#### Composition of the data monitoring committee, its role, and reporting structure {21a}

This is not applicable as described above.

#### Adverse event reporting and harms {22}

All participants are encouraged to contact the principal investigator or the RAND IRB to report complaints or adverse events (AE). Instructions for reporting AEs, serious adverse events (SAE), and complaints, as well as for contacting the investigators are included in the eConsent process and on all email communications provided to participants through the course of the study.

AEs and SAEs may be identified by IBCLCs during telelactation visits and during semi-structured interviews. They may also be reported by participants outside of synchronous encounters (e.g., emailed to the study team). Pacify will report AEs and SAEs detected during telelactation visits to the principal investigator and RAND’s IRB within 24 h. Events they report include situations where a breastfeeding parent or infant was referred to the emergency department (e.g., in the case of severe mastitis, mental health crisis) as well as cases in which abuse or neglect is observed. Pacify also reports these situations to local authorities as required.

We will report all AEs and SAEs to the IRB per RAND regulations. The project manager will prepare the appropriate IRB documentation, and will review with the principal investigator and determine severity and relation to study participation. All AEs and SAEs will also be reported to NIH within 48 h of occurrence.

An events summary will be sent to both RAND’s IRB and NIH as part of the final progress report. The report will include information on participants; demographic characteristics, recruitment, retention, adverse events, serious adverse events, and any significant changes to the research procedures that affect the safety of human subjects.

#### Frequency and plans for auditing trial conduct {23}

An appointed member of study team with experience in lactation research intermittently monitors study results and adverse event data. To allow effective monitoring, the monitor is provided with reports which include subject enrollment, subject retention, reasons for dropping out (if known), and a listing of all adverse events (AEs) that are plausibly related to the intervention or study procedures. Reports are provided to the monitor at 4-month intervals; however, AEs that are considered directly related to the intervention or other aspect of study participation are reported immediately to the monitor, the IRB, and NIH. After review of periodic reports, the monitor may ask for clarification or additional information from the PI. After such information is provided, the monitor makes a recommendation regarding the continuation, modification, or termination of the study. Communications from the monitor is shared with the IRB and NIH.

#### Plans for communicating important protocol amendments to relevant parties (e.g., trial participants, ethical committees) {25}

Summaries of study monitoring activities are provided to the IRB and NIH at the time of annual renewal. Further, relevant IRB actions are reported to NIH (e.g., amendments to the protocol) within 72 h if significant and as part of routine annual reporting if minor.)

#### Dissemination plans {31a}

The primary dissemination strategy is to produce multiple peer-reviewed publications and present findings at national scientific meetings, conferences, and webinars. We will work closely with the RAND Office of External Affairs (OEA), whose job is to ensure the wide dissemination of RAND reports and findings to the media and the public and to conduct targeted dissemination. OEA provides information and briefings to members of Congress as well as state and local decision makers, secures media coverage for reports and RAND experts, and leverages an array of communications strategies to raise the visibility of our projects, reports, and recommendations.

## Discussion

This protocol describes the first fully powered randomized study evaluating the impact telelactation on breastfeeding outcomes. Together, these aims will inform policy about reimbursement of telelactation and will identify strategies to further tailor these services to the needs and preferences of Black and Latinx parents who have historically had lower breastfeeding rates and reduced breastfeeding support. By conducting a digital trial, we anticipate recruiting a national sample representing diverse communities more rapidly and efficiently than through traditional methods [41], and by promoting a breastfeeding support intervention within a popular pregnancy app, this intervention is easily scalable and positioned to reach parents and infants at greater risk for limited breastfeeding and associated poor health outcomes.

This trial has a number of limitations. First, our recruitment method may not reach communities and individuals without reliable access to technology and digital literacy. Second, digital trials can face challenges with respect to fraudulent enrollment and attrition. Although we have incorporated robust strategies to address these challenges, it is unclear how effective they will be.

This research aims to improve children’s health through increased provision of human milk and reduce disparities in key maternal and child health outcomes. It will also inform the design and execution of future digital trials among pregnant and postpartum parents across minoritized groups.

### Trial status

This protocol is Version 1 (09.12.2021). Recruitment began on July 7, 2021, and will be completed in October 2023.

## Abbreviations

AE: Adverse event
IBCLC: International Board Certified Lactation Consultant
IRB: Institutional Review Board
ITT: Intention-to-treat
IV: Instrumental variable
NIH: National Institutes of Health
OEA: Office of External Affairs
RCT: Randomized controlled trial
SAE: Severe adverse event
Tele-MILC: Telehealth to Increase Mothers’ Lactation Confidence
